# Defining spinal motor neuron subtypes across development: from embryonic specification to postnatal maturation

**DOI:** 10.3389/fncel.2026.1819322

**Published:** 2026-06-18

**Authors:** Olga Blauth, Urszula Sławińska, Małgorzata Zawadzka

**Affiliations:** Laboratory of Neuromuscular Plasticity, Nencki Institute of Experimental Biology, Polish Academy of Sciences, Warsaw, Poland

**Keywords:** developmental specification, lower motor neuron, motor neuron subtypes, postnatal maturation, spinal motor neurons

## Abstract

Spinal motor neurons are essential for translating neural activity into coordinated muscle contraction, yet defining their functional subtypes across development remains a persistent challenge. While embryonic patterning establishes the initial positional and molecular framework of motor neuron identity, substantial refinement continues during early postnatal life as intrinsic electrophysiological properties, synaptic connectivity, and neuromuscular interactions mature. A major limitation in the field is the lack of temporally stable and functionally validated molecular markers that can reliably distinguish motor neuron subtypes across developmental stages, particularly during neonatal maturation when subtype-specific physiological features are emerging. In this review, we synthesize classical developmental studies with recent advances in single-cell transcriptomics, chromatin accessibility profiling, and multimodal approaches linking gene expression with electrophysiological and anatomical features. Focusing on lumbar spinal motor neurons that underlie locomotor behavior, we discuss how transcriptional programs, activity-dependent mechanisms, and non-cell-autonomous signals converge to shape subtype-specific maturation trajectories. We propose that motor neuron subtype identity is best understood as a dynamic molecular and physiological state shaped by developmental timing, circuit context, and activity-dependent mechanisms, rather than as a fixed category defined by a single marker. From this perspective, early postnatal life represents a sensitive window of identity consolidation during which molecular programs and functional properties become aligned. Establishing temporally robust subtype markers and integrating molecular and physiological datasets will be essential for resolving motor neuron diversity and for improving our understanding of subtype-selective vulnerability in neuromuscular diseases. While this review emphasizes embryonic and early postnatal development, understanding how molecular subtypes stabilize in the adult spinal cord, despite ongoing activity-dependent physiological plasticity, remains an essential reference point for defining temporally robust motor neuron identities.

## 1 Introduction

Motor neurons (MNs) are highly specialized cells that transmit neural commands from central circuits to skeletal muscles, thereby initiating and modulating muscle contractions and movement. During embryogenesis, MNs acquire their initial identities through tightly regulated transcriptional programs that establish motor pools, axonal trajectories, and target specificity. Although these early developmental processes lay the foundation for motor circuit formation, MN differentiation does not conclude at birth.

Indeed, while the pace of MN maturation slows markedly after birth, substantial refinement of molecular identity, intrinsic electrophysiological properties, and circuit integration continues throughout the postnatal life (Vinay et al., 2000a; Stifani, 2014; Hadders-Algra, 2018). Despite its importance, the molecular mechanisms governing postnatal MN maturation and subtype-specific specification remain incompletely understood. A major challenge in the field is the lack of a consensus set of temporally stable and functionally validated molecular markers that consistently distinguish MN subtypes across developmental stages, particularly during early postnatal development.

This limitation has important functional and clinical implications. Accumulating evidence indicates that distinct MN subtypes exhibit differential vulnerability to injury, degeneration, and regenerative failure, underscoring the need for subtype-resolved molecular characterization (Ovsepian et al., 2024). This knowledge gap impedes our understanding of fundamental developmental processes and, consequently, constrains efforts to elucidate the molecular mechanisms underlying severe neurodegenerative disorders, such as amyotrophic lateral sclerosis (ALS) and spinal muscular atrophy (SMA), both of which are associated with the dysfunction and degeneration of MNs.

Recent advances in transcriptomic profiling, chromatin accessibility analyses, and *in vivo* electrophysiology provide unprecedented opportunities to link MN molecular identity with intrinsic electrical properties and circuit function. These approaches challenge classical classification schemes based solely on anatomy or single molecular markers and, instead, reveal MN diversity as a dynamic, continuously evolving process. However, despite these advances, translating emerging molecular signatures into stable subtype definitions across developmental time remains challenging. Many commonly used markers are transiently expressed, shared among multiple subtypes, or appear only after key functional properties have already been established. This limitation significantly constrains efforts to map molecular identity onto physiological specialization, circuit integration, and selective vulnerability in disease.

In this review, we integrate foundational developmental studies with recent molecular and physiological insights to propose a framework in which MN subtypes are best understood as dynamic molecular-electrophysiological states that emerge progressively over development. We focus particularly on maturation and subtype specification of lumbar spinal MN, which are located in the ventral horn of the spinal cord and project axons through peripheral nerves to innervate hindlimb muscles. We synthesize how transcriptional programs, extracellular signaling, adhesion molecules, and activity-dependent mechanisms converge to establish precise molecular, anatomical, and functional diversity of MN subtypes and describe technological advances that enable subtype-resolved analysis. Finally, we discuss how developmental and maturation processes shape selective vulnerability of distinct MN subtypes in neuromuscular disease, highlighting the key questions that must be addressed to advance therapeutic strategies aimed at preserving or restoring MN function.

## 2 Defining motor neuron subtypes: functional organization and limitations of molecular markers

Motor neurons are broadly classified as upper motor neurons (UMNs) and lower motor neurons (LMNs) based on the location of their cell bodies and projection targets. UMNs reside in the premotor and primary motor cortex and form glutamatergic connections exclusively with LMNs and interneurons. Lesions affecting UMNs typically result in spasticity, hyperreflexia, and impaired voluntary motor control, reflecting disruption of descending motor commands rather than loss of direct muscle innervation (Stifani, 2014). Importantly, UMNs exert powerful modulatory control over spinal motor circuits, shaping MN recruitment, gain, and coordination during movement. Alterations in this descending control can profoundly influence MN functional states without directly affecting their survival.

LMNs are located in specific brainstem nuclei and in the ventral horn of the spinal cord (Javed and Daly, 2023). In contrast to UMNs, they extend axons outside the CNS to directly innervate peripheral targets. LMNs are cholinergic and receive inputs from UMNs, sensory neurons, and interneurons. LMN lesions result in paralysis, as no alternative pathway exists to convey motor commands to the muscle (de Carvalho and Swash, 2016). LMNs are further classified into branchial, visceral, and somatic subtypes. This review focuses specifically on somatic LMNs, which innervate skeletal muscles and are central to voluntary motor control (de Carvalho and Swash, 2016).

The classification of spinal MNs into distinct functional subtypes is fundamental for understanding how the nervous system controls muscle activity, coordinates movement, and determines patterns of selective vulnerability in diseases. Within the spinal cord, MNs are broadly divided into alpha, beta, and gamma subtypes, based on their target innervation patterns and functional roles in regulating muscle force and proprioceptive feedback. During development, MNs that share peripheral targets and receive common afferent inputs assemble into anatomically discrete clusters, known as motor pools, which provide the structural basis for precise and task-specific motor output (Price et al., 2002). Early hypotheses proposing clonal relationships between MNs and their muscle targets have not been supported by lineage-tracing studies, which demonstrate that individual progenitors generate diverse neuronal and glial cell types (Moody and Jacobson, 1983; Leber et al., 1990).

Alpha (α) motor neurons, which innervate extrafusal muscle fibers and initiate muscle contraction, can be further divided into slow (S), fast fatigue-resistant (FR), and fast fatigable (FF) subtypes. Anatomically, alpha MNs are characterized by a large cell body and a well-characterized neuromuscular junction (Burke et al., 1973; Stifani, 2014). They receive thousands of synaptic inputs, both excitatory and inhibitory, from descending fibers, local interneurons, and sensory afferents (Sengul and Watson, 2012; Khan and Marquardt, 2024). During early postnatal development, α MNs acquire subtype-specific firing patterns, with immediate firing associated with slow muscle units and delayed firing linked to fast units, indicating that functional specialization emerges progressively rather than being fully specified at birth (Lee and Heckman, 1998; Russier et al., 2003; Leroy et al., 2014). Despite extensive characterization of α MN physiology, the molecular mechanisms that stabilize these subtype-specific properties during postnatal maturation remain incompletely understood.

In contrast to α MNs, beta (β) MNs have received comparatively little attention, despite their unique dual innervation pattern of both extra- and intrafusal muscle fibers. Previously thought to be restricted to lower vertebrates, β MNs are known to be present in mammals, where they constitute a substantial fraction of motor units and innervate the majority of muscle spindles (Manuel and Zytnicki, 2019). Functionally, β MNs are divided into static and dynamic subclasses based on the intrafusal fibers they innervate. Static β MNs innervate nuclear chain fibers and increase the firing rates of both type Ia and type II sensory afferents at a given muscle length, thereby enhancing tonic sensory signaling. In contrast, dynamic β MNs innervate nuclear bag fibers, where their activation increases the stretch-sensitivity of Ia afferents by stiffening these fibers. Through these complementary mechanisms, β MNs provide an additional layer of control over proprioceptive feedback while contributing directly to muscle force generation (Manuel and Zytnicki, 2011; Stifani, 2014). Current evidence suggests that the functional role of β motor neurons in motor control is still incompletely characterized, which may partly stem from the lack of well-established molecular markers.

Gamma (γ) motor neurons innervate intrafusal muscle fibers within muscle spindles and regulate proprioception. Phylogenetically, γ MNs are best developed in mammals, whereas lower vertebrates (e.g., amphibians) use a β-skeletofusimotor system alone to control the sensitivity of their muscle spindles (Shneider et al., 2009). γ MNs comprise roughly 30% of the motor pool, typically have smaller somata than α motor neurons, have simpler and less branched dendritic trees, and do not receive group Ia monosynaptic input nor C- buttons (Arvidsson et al., 1987; Kanning et al., 2010; Manuel and Zytnicki, 2019). Functionally, γ MNs can fire at increased rates, are more excitable than α MNs, and have other electrophysiological differences that likely vary based on age and species (Wilkinson, 2021). Their firing increases the tension of intrafusal muscle fibers and therefore mimics the muscle stretch. Like β MNs, γ MNs are functionally divided into two subtypes: static, innervating nuclear chain fibers and static nuclear bag fibers, and dynamic, innervating the dynamic nuclear bag fibers. Although their functional role is well established, the intrinsic biophysical mechanisms that support their early recruitment and high firing gain remain incompletely understood. This limited insight largely reflects their small size, which complicates *in vivo* electrophysiology, as well as the historical reliance on early postnatal *in vitro* preparations, where spinal circuits are still immature (Kang et al., 2024; Sharples et al., 2025). Recent methodological advances in identifying γ MNs within more mature spinal cord preparations might open new opportunities to investigate their intrinsic properties across development.

Most motor pools contain both α, β, and γ MNs, which arise from common progenitors and then differentiate to form specific cell types that differ in morphology, physiology and connectivity (Prochazka, 1996; Shneider et al., 2009). The investigation into the mechanisms driving the differentiation of α, β, and γ MNs has been hindered by the absence of specific molecular markers to distinguish these functionally distinct populations during development. In neonates, this challenge is particularly evident since MN subtypes are morphologically similar, and classification has therefore relied largely on physiological criteria such as conduction velocity (Simon et al., 1996; Manuel and Zytnicki, 2019).

In adults, γ and α MNs can be distinguished by the expression of the transcription factor Errg (Estrogen Related Receptor Gamma). However, expression is initially broader during early postnatal development and becomes progressively restricted to γ MNs over the first two postnatal weeks (Friese et al., 2009). γ MNs might be identified based on their lower expression of the pan-neuronal nuclear protein NeuN (Shneider et al., 2009; Misawa et al., 2012). Unfortunately, the usefulness of NeuN appears to be limited, as its immunoreactivity in neurons may decrease after injury, potentially indicating altered neuronal integrity or changes in protein expression associated with degeneration or stress responses (Unal-Cevik et al., 2004; Santamaría et al., 2023). Recently, Prdm16 and Mecom have been proposed as early markers of primary and fast secondary MNs in zebrafish (D'Elia et al., 2023). However, the differences between aquatic and terrestrial motor systems limit direct extrapolation to mammals, particularly given the absence of muscle spindles- and thus γ and β MNs-in fish (Butler, 1980; Ito et al., 1992). Gfrα1 (GDNF receptor) has also been proposed as a potential early molecular marker for γ MNs at birth (Shneider et al., 2009).

Distinguishing specific MN populations is easier in adult animals than in neonates, as several subtype-specific gene markers are well established, and the neurons themselves differ in size and conduction properties. Although advances in genetics and transcriptomics have expanded the catalog of MN markers, significant limitations remain widely used. Recent studies have demonstrated limitations of pan-MN markers such as ChAT (choline acetyltransferase), VAChT (vesicular acetylcholine transporter), and Mnx1 (motor neuron and pancreas homeobox 1). Mnx1 expression declines sharply after birth, ultimately approaching near-undetectable levels, thereby complicating postnatal studies (Patel et al., 2022; Lowry et al., 2025). In adult rodents, α MNs express markers such as NeuN and osteopontin, with additional markers distinguishing fast and slow subtypes, whereas γ MNs are characterized by low NeuN expression and selective expression of Gfrα1, serotonin receptor 5-HT1d, and Esrrg (Friese et al., 2009.; Shneider et al., 2009; Chakkalakal et al., 2010; Manuel and Zytnicki, 2011; Misawa et al., 2012; Khan and Marquardt, 2024).

Within the adult α MN population, additional subtype-specific markers can also be identified: FF MNs express Calcitonin gene-related peptide (CGRP/Calca), Chondrolectin (Chodl), Delta-like homolog 1 (Dlk1), and Matrix metallopeptidase 9 (MMP-9) (Manuel and Zytnicki, 2019). Several gene markers have also been identified for S MNs - the synaptic vesicle protein SV2a is expressed postnatally in the presynaptic terminals of S MNs, which innervate type I and small type IIA muscle fibers (Chakkalakal et al., 2010). Also, Potassium Calcium-Activated Channel Subfamily N Member 3 (SK3), Estrogen Related Receptor Beta (Errb) are expressed in S MNs (Manuel and Zytnicki, 2019; D'Elia et al., 2023).

Together, the current classification scheme appears far from complete in capturing the full diversity of MN subtypes and their context-dependent gene expression profiles, particularly during early postnatal life. The incompleteness and temporal instability of available markers underscore the need for integrated genetic, transcriptomic, and physiological approaches to define MN subtypes more precisely. Resolving these challenges will be essential for understanding how developmental programs and postnatal maturation progressively link the molecular identity of motor neurons to their differentiation timing, functional specification, and selective vulnerability to stressors.

## 3 Early programs of motor neuron specification: patterning and commitment

Motor neuron development is a tightly orchestrated process that integrates molecular patterning, spatial organization, timed differentiation, and axonal navigation to establish functional neuromuscular circuits. Defining when and where MNs are born is essential for understanding early developmental programs that shape their specification and postnatal maturation.

**Figure 1:**
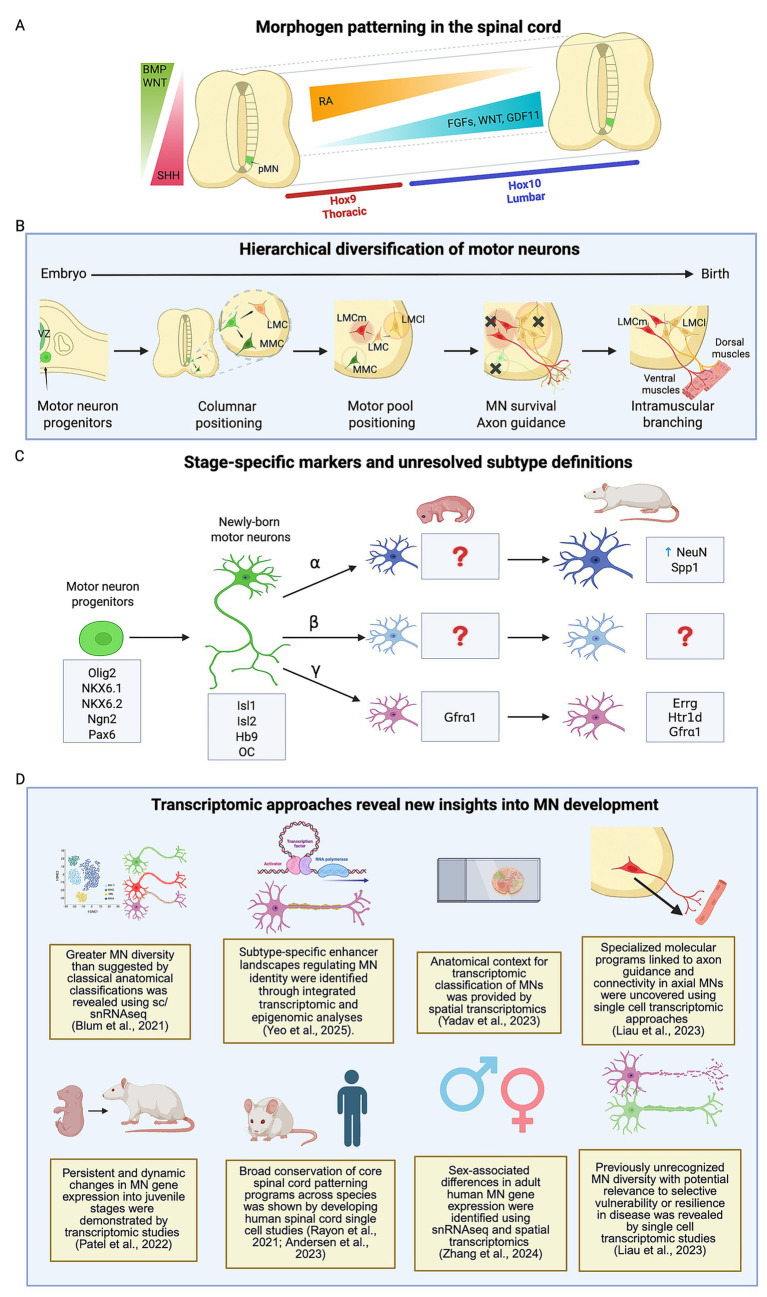
Developmental assembly and diversification of spinal motor neuron subtype identity. **(A)** Morphogen patterning in the spinal cord. Schematic representation of dorsoventral and rostrocaudal patterning of the ventricular zone during early spinal cord development. Ventral identity is specified by Shh signaling from the notochord and floor plate, opposed by dorsal BMP and Wnt gradients. Along the rostrocaudal axis, RA and FGF signaling regulate Hox-dependent segmental identity, with Gdf11 contributing to caudal specification. Integration of these gradients establishes the pMN progenitor domain and positional coordinates for motor neuron generation. **(B)** Hierarchical diversification of motor neurons. Developmental progression from pMN progenitors to mature motor neuron subtypes. Newly born motor neurons first undergo columnar organization, followed by motor pool specification linked to target muscle identity. Subsequent maturation includes programmed cell survival, axon guidance, and intramuscular branching, progressively refining functional specialization. **(C)** Stage-specific markers and unresolved subtype definitions. Early lineage stages are defined by well-established markers. By contrast, molecular signatures distinguishing neonatal α-, β-, and γ-subtypes remain incomplete, with limited and non-universal marker sets and particularly poor resolution of β-motor neurons. Although adult functional classes are established physiologically, their comprehensive molecular definition is still evolving. **(D)** Transcriptomic approaches reveal new insights into MN development. Sc/snRNA-seq, spatial transcriptomics, and integrated transcriptomic-epigenomic analyses have expanded current understanding of MN diversity, maturation, and subtype identity. These approaches revealed greater molecular heterogeneity than suggested by classical anatomical classifications, identified subtype-specific regulatory programs and enhancer landscapes, linked transcriptomic MN classes to spatial organization in the spinal cord, and showed that MN gene expression remains dynamic into juvenile stages. They also provide new insight into species-conserved developmental programs and subtype-specific features potentially relevant to selective vulnerability or resilience in disease.

In rodents, MNs are generated between embryonic day (E) 11–12 in the cervical spinal cord and E13-14 in the lumbo-sacral cord in rats, whereas at E9-11 in mice (Altman and Bayer, 1984). MNs arise from neuroepithelial precursors in the ventricular zone of the neural tube (VZ) influenced by overlapping gradients of morphogens that pattern progenitor identity along the dorsoventral (DV) and rostrocaudal (RC) axes (Hamburger, 1977). Key signaling sources include Sonic hedgehog (Shh) secreted from the notochord and floor plate, bone morphogenetic proteins (BMPs) and Wnts from the roof plate and ectoderm, retinoic acid (RA) from paraxial mesoderm, fibroblast growth factors (FGFs) from the caudal mesoderm and tail bud, and growth differentiation factor 11 (GDF11), which contributes to rostrocaudal patterning of the spinal cord. Together, these signals establish discrete progenitor domains of the VZ that give rise to distinct neuronal lineages (Figure 1).

Shh signaling is essential for ventral spinal cord patterning and for specification of the pMN domain, from which MNs and several ventral interneuron classes (V0–V3) arise. Disruption of Shh signaling results in loss or severe reduction of MNs and multiple ventral interneuron populations, underscoring its instructive role in ventral identity (Novitch et al., 2003). RA and FGF signaling further refine pMN progenitor identity by regulating the timing of differentiation and RC patterning (Novitch et al., 2003). Although RA is well established as a caudalizing signal during development, studies in regenerative model organisms such as axolotls suggest that it may also regulate progenitor proliferation and patterning during regeneration (Khan and Marquardt, 2024). Although the role of Shh in promoting ventral neural identities is well established, the contributions of BMP and Wnt signaling to dorsal spinal cord patterning remain less clearly defined (Sagner and Briscoe, 2019).

Mechanistically, morphogen gradient signals are implemented through transcriptional gene regulatory networks. Along the DV axis, Shh signaling regulates Gli transcription factors, which control the expression of class I and class II homeodomain transcription factors. Reciprocal repression between these factors (e.g., Pax6 versus Nkx6.1/Nkx2.2) stabilizes progenitor identities and restricts lineage potential (Briscoe et al., 2000; Novitch et al., 2001, 2003; Shirasaki and Pfaff, 2002). Co-expression of Pax6 and Nkx6.1 induces Olig2, the key transcription factor defining the pMN domain (Novitch et al., 2001). Olig2 then activates the pro-neural gene Ngn2 while repressing premature expression of post-mitotic MNs factors such as Isl1, Lhx3, and Mnx1 (Hb9) (Lee et al., 2005; Thaler et al., 2002). As Ngn2 level rise, they overcome Olig2-mediated repression, driving cell-cycle exit and neuronal differentiation. Also, GDE2, expressed by newly born MNs further promotes differentiation through cell-autonomous mechanisms (Francius and Clotman, 2014). This balance ensures that progenitors are primed for neurogenesis without differentiating prematurely.

As pMNs transit to a post-mitotic state, their transcriptional program undergoes a coordinated shift. The proneural factor Ngn2 is replaced by the bHLH transcription factors NeuroM and NeuroD, which promote neuronal differentiation. At the same time, LIM homeodomain transcription factors Isl1 and Lhx3/4 form a ternary complex with NLI (Ldb/Clim) that cooperates with bHLH proteins to activate motor neuron-specific genes, including Mnx1 (Hb9) (Lee et al., 2005). Concurrent downregulation of Olig1/2 marks the commitment to terminal differentiation. As newly generated MNs stabilize their transcriptional identity, they begin to express post-mitotic markers and initiate axon extension toward peripheral muscle targets (Santiago and Bashaw, 2014). Importantly, these embryonic transcriptional programs establish an initial positional and molecular framework of MN identity rather than a fully mature phenotype, which is subsequently refined through migration, circuit integration, and postnatal activity.

## 4 From specification to spatial organization: motor neuron migration and positioning

Precise positioning of MN cell bodies within the spinal cord is fundamental for the assembly of functional motor circuits. Emerging evidence suggests that spinal neurons migrate along radial glial scaffolds in a manner analogous to the mechanism that drives cortical neurons’ migration. Spinal radial glia express the Reelin adaptor protein Dab1 and are exposed to Reelin signaling, which, together with Notch signaling, limits aberrant MNs displacement (Lee and Song, 2013). Although MN identity specification and axon guidance have been extensively studied, the processes that govern somatic positioning within the spinal cord have received far less attention (Kim et al., 2019).

MN precursors originate adjacent to the floor plate, where exposure to a graded of Shh signal initiates MN-specific transcriptional programs. Upon exiting the cell cycle, newly born MNs detach from the ventricular zone and migrate laterally. This process is promoted by Foxp2 and Foxp4, which repress cadherin-2 (Cdh2), reducing adhesion to the neuroepithelium and linking transcriptional regulation to changes in cell–cell adhesion and membrane dynamics (Kim et al., 2019). Overall, the emergence of newly born MNs from the pMN domain depends on a tightly regulated balance between proliferation and differentiation. While Olig2 and Ngn2 play central roles in fate specification, additional mechanisms are necessary to stabilize the MN phenotype. Once their general identity is established, MNs undergo further differentiation to acquire the specialized features required for their function (Stifani, 2014). These events highlight how fate specification and migration are tightly coupled rather than sequentially independent processes.

Motor neuron migration during spinal cord development can be conceptually divided into three sequential phases (Jessell, 2000). The first phase, columnar positioning, involves the movement of post-mitotic MNs from the pMN domain toward their respective longitudinal positions within the spinal cord columns. The second phase, motor pool positioning, refines MN organization within each column into discrete motor pools that correspond to specific muscle targets. The third phase, axon exit and peripheral trajectory selection, occurs in parallel with somatic positioning: newly differentiated MNs extend axons toward the ventral root, traverse the CNS-PNS boundary, and initiate target-directed growth in response to coordinated guidance cues. Together, these phases ensure that MN cell bodies are precisely arranged to establish functional neuromuscular circuits. It is important to recognize that these processes are not strictly temporally or mechanistically segregated. Instead, they exhibit extensive overlap, with aspects of motor pool refinement commencing even as columnar positioning is still being established. This overlap underscores the integrated nature of MN differentiation, migration, and connectivity, and highlights a key unresolved challenge: how early transcriptional programs interface with cell biological mechanisms to ensure precise MN positioning and stable circuit assembly?

### 4.1 Columnar positioning

Spinal MNs become positioned into columns at specific mediolateral locations within the neural tube. This columnar arrangement is essential for establishing proper axonal trajectories and forming accurate neural circuits. In the lumbar spinal cord, several motor neuron columns are distinguished, among them the lateral motor columns (LMC) and the median motor column (MMC). LMC columns, which innervate limb muscles, are located in both the brachial and lumbar regions (Guthrie, 2004). Lumbar LMC neurons innervate hindlimb muscles and are subdivided into the medial (LMCm) and lateral (LMCl) divisions, which later give rise to distinct motor pools. MMC, existing throughout the thoracic and lumbar regions, innervates axial muscles and, in lumbar segments, provides a positional scaffold for organizing neighboring limb-innervating LMC neurons. A smaller population of hypaxial motor column (HMC) neurons, although primarily associated with thoracic segments, is also detected in the lumbar region; however, its molecular identity and functional contribution remain less well defined (Francius and Clotman, 2014; Stifani, 2014). Collectively, these columns generate a precise mediolateral map in the lumbar spinal cord, enabling accurate connectivity with peripheral targets. Recent single-cell and single-nucleus transcriptomic studies have revealed substantial heterogeneity among spinal MNs compared to what was previously appreciated, with multiple molecularly distinct subtypes present even within a “classical” column. In the human spinal cord, novel column- and subdivision-specific gene expression patterns have been identified: TAC1, NRP1, and NTRK2 are enriched in the LMC; TSHZ2, DUSP4, and ID4 are specific to LMCm; and PCDH9, DPP6, and OPRK1 are enriched in LMCl neurons (Shi et al., 2024). These findings suggest that traditional column classification provides only a coarse framework, and future efforts should integrate transcriptomic, connectivity, and functional data to delineate subpopulations of MNs more precisely (Milich et al., 2021; Blum and Gitler, 2022; Liau et al., 2023). Moreover, chromatin accessibility and transcription factor occupancy studies indicate that epigenomic state in some contexts predicts MN subtype identity and vulnerability more robustly than steady-state gene expression alone. Consequently, subtype-specific transcriptional markers may reflect downstream consequences of a permissive or restrictive chromatin landscape rather than primary determinants of identity (Milich et al., 2021; Shi et al., 2024; Gauberg et al., 2025).

Generation of MNs in the spinal cord follows a precise spatiotemporal sequence, neurons located more ventrally and rostrally being produced earlier (Stifani, 2014). Along the dorsoventral axis, Shh signaling from the notochord and floor plate induces the pMN progenitor domain, establishing the competence to generate MNs. Once MN progenitors are specified, RC patterning mechanisms determine their subtype identity. This RC patterning is governed by mesoderm-derived signals, including fibroblast growth factors (FGFs), growth differentiation factor 11 (GDF11/BMP-11), retinoic acid (RA), and other TGF-β family members, which act in a concentration-dependent manner to regulate the expression of homeobox (Hox) transcription factors (Bel-Vialar et al., 2002; Stifani, 2014). The resulting Hox expression profile in post-mitotic MNs defines their columnar identity (Bel-Vialar et al., 2002; Guillemot and Zimmer, 2011).

In the lumbar spinal cord, MN positional identity is established by combinatorial Hox-c expression, refined through extrinsic signals such as FGFs, RA, and GDF11. This Hox-c code directs the formation of caudal motor columns, with Hox10 paralogs playing a central role in the generation of LMC neurons (Liu et al., 2001; Rousso et al., 2008). The molecular mechanisms specifying LMC neurons mirror those that define limb position along the body axis, highlighting the shared roles of FGFs and Hox proteins in limb-level patterning (Dasen et al., 2003; Rousso et al., 2008). Once LMC neurons are matched to their limb targets, Hox proteins further refine MN topography by linking RC identity to dorsoventral axonal trajectories (Dasen et al., 2003). Recent studies have expanded this “Hox-centered” view by identifying Irx/Iro homeobox genes as evolutionarily conserved regulators of MNs diversification. Functional analyses show that Irx2 promotes LMC specification, whereas Irx6 restricts LMC identity in rostral brachial segments, indicating complementary and region-specific functions. Importantly, Irx genes act downstream of Hox proteins, positioning them as mediators that translate RC patterning cues into MN subtype specification (Catela et al., 2025).

Beyond transcriptional control, extracellular signaling pathways further refine motor neuron subtype composition and contribute to the diversification of specific motor columns. GDE2 promotes the generation of specific MN subtypes by inhibiting Notch signaling, thereby influencing the differentiation and final columnar composition (Sabharwal et al., 2011). For instance, the identity of medial motor column (MMC) neurons depends on Wnt signaling and expression of the Prdm family transcription factor Mecom, whereas molecular programs specifying other motor columns, such as the hypaxial motor column (HMC) remain largely uncharacterized (Khan and Marquardt, 2024).

### 4.2 Motor pool positioning

Motor pools are spatially and functionally distinct clusters of MNs that innervate specific muscle groups. Their precise positioning within the spinal cord ensures accurate connectivity with peripheral targets and is critical for coordinated motor behavior. Understanding the molecular and cellular mechanisms that guide MN migration and pool segregation is essential for elucidating the assembly of spinal motor circuits. Although this process is well described, the molecular and cellular programs that guide MN migration to the ventral horn and direct their sorting into distinct pools remain incompletely understood (Dewitz et al., 2018). To bridge general MN migration with motor pool formation, it is important to consider how LMC neurons first establish divisional identity before undergoing secondary clustering.

Within the LMC, neurons undergo secondary clustering into motor pools, sharing intrinsic characteristics and distinct axon projection patterns (Dewitz et al., 2018; Gauberg et al., 2025). In the lateral motor column (LMC), motor pool formation follows a hierarchical organization: medial LMC (LMCm) neurons are generated first, providing a scaffold for later-born lateral LMC (LMCl) neurons, which migrate outward to establish a discrete pool (Landmesser, 2001). The early-born LMCm neurons rapidly induce Raldh2 expression and become a local source of RA, a signal that, together with RA from the paraxial mesoderm, drives a second wave of neurogenesis that produces the lateral LMCl subdivision. The observation that even LMC neurons lacking proper LMCm identity can still activate Raldh2 underscores that RA production is an intrinsic feature of post-mitotic LMC neurons rather than a property of a single columnar subset. RA, in turn, is required for LMC specification and for the long-term survival of LMC neurons, a process further stabilized by Hox-dependent expression of RARβ (Vermot et al., 2005; Sabharwal et al., 2011; Francius and Clotman, 2014; Lee et al., 2023).

The medial-lateral division of the LMC is consolidated by a cross-repressive transcriptional switch between Isl1 and Lhx1, which assigns medial versus lateral identity and directs neurons to their proper settling positions. Isl1 promotes LMCm fate, including medial settling and ventral limb trajectories, whereas Lhx1 drives LMCl differentiation, lateral positioning, and dorsal axon guidance (Francius and Clotman, 2014; Lee et al., 2023). Additional transcriptional regulators refine this process: in LMCl neurons, Foxp1 and Lhx1 cooperate to maintain high levels of the Reelin-signaling intermediate Dab1, which is required for their proper lateral settling within the LMC but does not influence their axonal projection patterns (Palmesino et al., 2010). Onecut (OC) factors, in turn, support Isl1 expression and LMCm identity; The discrepancy between LMCl expansion and the lack of lateral displacement in OC mutants suggests that additional, yet unidentified, downstream effectors contribute to the stabilization of columnar architecture (Lee et al., 2023), a notion further supported by the observation that ablation of Isl2 leads to dispersion of motor pools, predominantly affecting the median motor column (MMC) and lateral motor column (LMC) populations, resulting in disorganized MN soma arrangement (Catela et al., 2025).

Beyond transcriptional regulation, adhesion molecules coordinate migration and spatial organization of MNs. Classical cadherins and their associated catenin partners have emerged as key regulators of MN positional organization. N-cadherin, a type I cadherin expressed by all MNs plays dual roles: it controls the dorsoventral columnar position of neurons in the ventral horn and contributes to the mediolateral positioning and segregation of LMCl neurons. Loss of N-cadherin signaling shifts LMC neurons to settle too ventrally, but the relative arrangement of motor pools along the dorsoventral axis is largely preserved, suggesting that additional β-/γ-catenin-dependent mechanisms contribute to dorsoventral pool clustering (Demireva et al., 2011). Consistent with this, genetic elimination of N-cadherin or disruption of all classical cadherin function via loss of β- and γ-catenins further disrupts divisional segregation and pool clustering in mouse embryos (Demireva et al., 2011; Dewitz et al., 2018).

Catenin-dependent adhesion also cooperates with nectins, a family of calcium-independent immunoglobulin-like adhesion molecules, through direct interaction with the cytosolic adaptor protein afadin. Nectin/afadin interactions fine-tune adhesive properties among developing neurons. Afadin is essential for spinal morphogenesis, and its loss disrupts gait and interneuron wiring without affecting pool segregation, indicating pool-specific organization relies on catenin-dependent, afadin-independent pathways (Takai and Nakanishi, 2003; Skarlatou et al., 2020). Radial migration and lateral displacement of LMCl neurons require both cadherin/catenin signaling and afadin-independent mechanisms, emphasizing their coordinated role in establishing layered LMC architecture (Takai and Nakanishi, 2003; Demireva et al., 2011; Dewitz et al., 2018; Skarlatou et al., 2020).

Hox proteins assign both rostro-caudal identity and intra-segmental diversity; disruptions reroute motor pool fate and axonal trajectories. Downstream of Hox activity, post-mitotic Nkx6.1 is required for the specification of particular adductor-associated pools, whereas PEA3/Etv4 governs the identity of pools innervating muscles such as the cutaneous maximus and latissimus dorsi (Francius and Clotman, 2014; Gauberg et al., 2025). Notably, PEA3 activation requires GDNF signaling from peripheral muscle, and HGF-Met signaling further expands PEA3 expression non-cell-autonomously, linking muscle-derived cues to central pool identity (Haase et al., 2002). Pool-specific expression of type II cadherins and semaphorins subsequently mediates segregation and clustering, enabling neurons with shared molecular signatures to consolidate into discrete, spatially coherent motor pools (Cohen et al., 2005).

In summary, motor pool positioning emerges from the integration of temporal neurogenesis, RA-dependent columnar specification, cross-repressive transcription factor networks, adhesion-mediated migration, Hox-patterned pool determinants, and finely tuned extrinsic signals. Together, these mechanisms generate the precise spatial and functional architecture required for limb-specific motor control.

## 5 Crossing the CNS-PNS boundary: motor axon exits and target-directed growth

Once motor neurons are organized within the spinal cord, they extend axons toward their peripheral targets, such as the muscles of the developing limb. Within the limb mesenchyme, axons further organize into distinct nerve trunks, each following precise trajectories determined by the identity of the originating MNs (Gauberg et al., 2025). The unique positioning of MNs at the CNS-PNS interface is shaped by structural cues, including boundary cap cells, the basal lamina, and radial glia (Lee et al., 2015). A critical decision involves whether axons cross the CNS-PNS boundary, ultimately shaping motor connectivity.

During embryonic development, MNs send their axons through motor exit points (MEPs) at sites populated by boundary cap (BC) cells (Bravo-Ambrosio and Kaprielian, 2011). Their strategic localization suggested a potential role in guiding motor axons through MEPs. However, neural crest ablation or BC cell loss does not disrupt axon pathfinding, but instead leads to ectopic MN soma migration, indicating that BC cells function primarily to retain MN cell bodies within the spinal cord rather than direct axonal trajectories (Bravo-Ambrosio and Kaprielian, 2011).

Axon pathfinding, like a neuronal migration, is orchestrated by attractive and repulsive guidance cues. A multiplicity of factors allows motor axons to leave the central nervous system, including mechanisms that prevent these axons from being attracted to inappropriate targets within the spinal cord (Suter and Jaworski, 2019). In this context, Robo signaling contributes to proper trajectory selection not only by mediating repulsion from Slit proteins but also by suppressing DCC-dependent attraction to Netrin-1 and thereby promoting a repulsive response to this cue (Nickerson et al., 2025). Importantly, recent transcriptomic studies indicate that these guidance programs are embedded within subtype-specific molecular identities. Single-cell profiling of embryonic spinal motor neurons revealed distinct MN populations enriched for axon-guidance and adhesion-related genes, suggesting that differences in pathfinding competence are coupled to emerging subtype programs rather than imposed solely by external cues. In particular, molecularly distinct axial MN subtypes defined by Satb2, Nr2f2, or Bcl11b expression exhibit different guidance-associated transcriptional signatures, consistent with the idea that intrinsic gene programs contribute to trajectory selection and target specificity (Liau et al., 2023). Several mechanisms prevent soma of MNs from overshooting their settling position and follow their axons into the periphery, including inhibitory interactions with BC, perineurial glia, and radial glia endfeet at MEPs, anchoring by the cell adhesion molecule TAG-1, signaling by secreted Semaphorins and Slits, and stabilization mediated by β- and γ-catenins through cadherin-based adhesion (Demireva et al., 2011; Radomska and Topilko, 2017; Suter et al., 2020; Nickerson et al., 2025). The MN cell surface protein TAG-1 anchors MN cell bodies in the spinal cord, mediates motor axon fasciculation during CNS exit, and guides them past dorsal root ganglia (Suter et al., 2020). After axon growth through MEPs, BC differentiate into Schwann cells, endoneurial fibroblasts, satellite glial cells, multiple subtypes of sensory neurons, terminal glia at sensory endings, and subepidermal glia (Suter and Jaworski, 2019).

Local disruption of the CNS-PNS boundary is critical for axon to cross the transition zones, but instructive cues are also required to guide axons toward and across this interface. In mice, the *CXCL12*-*CXCR4* signaling pathway plays a key role in motor axon exit: *CXCR4* is expressed by motor neurons, while *CXCL12* by the meninges and surrounding mesenchyme. In *Cxcl12* or *Cxcr4* mutants, many motor axons fail to leave the spinal cord, instead projecting medially to the ventricular zone or dorsally to the dorsal root entry zone (Lieberam et al., 2005). On the other hand, Slit-Robo signaling prevents ventral commissure crossing: the floor plate produces Slits, which act as repellents for motor axons, and deletion of *Robo1* and *Robo2* causes motor axons to inappropriately cross the midline (Nickerson et al., 2025). Recent studies indicate that NFIA contributes to the regulation of gene expression programs involved in motor axon guidance toward skeletal muscle targets during development (Gauberg et al., 2025).

Axon guidance of spinal motor neurons is orchestrated by a coordinated program of intrinsic transcriptional identity and extrinsic guidance cues that steer growth cones to specific muscle targets, where target-derived signals then refine survival and subtype-specific properties. Intrinsic factors such as Hox and LIM-homeodomain proteins (and downstream regulators like Foxp1) set up motor-column and pool identities and control the complement of axon guidance receptors expressed by motor neurons, thereby biasing particular axons toward medial versus lateral limb trajectories or toward specific muscles (Bonanomi and Pfaff, 2010). Growth-cone receptors (Ephs/ephrins, semaphoring-neuropilin/plexin complexes, netrins/DCC, Slits/Robos and others) interpret spatially patterned attractive and repulsive cues in the limb and trunk mesenchyme to generate the stereotyped branching and target selection of motor axons (Hollis, 2015). After peripheral contact, target-derived trophic factors, such as GDNF for fusimotor γ MNs, reinforce survival, molecular programs, and mature phenotypes (Gould et al., 2008). Early MN proliferation and migration are independent of limb development. However, in rats, motor axons enter the forming limb muscle masses around E13.5-E14.5, coinciding with the onset of myogenic differentiation and the appearance of early acetylcholine receptor (AChR) clusters on myotubes that indicate the establishment of the first neuromuscular contacts in the embryo (Hurren et al., 2015). Approximately 50% of MNs are eliminated during a critical window from E14 to P3, a process termed physiological cell death (Sendtner et al., 2000).

Upon reaching their muscle targets, motor axons encounter a postsynaptic landscape already partially organized: prior to innervation, neural acetylcholine receptor (AChR) clusters form in the central region of muscle fibers through a process of muscle-autonomous prepatterning that requires MuSK and rapsyn but is independent of neural agrin (Glass and Yancopoulos, 1997; Kim and Burden, 2008). Once nerve terminals arrive, neuronal agrin-secreted by the motor terminal-activates MuSK through its co-receptor LRP4, stabilizing and enlarging AChR clusters at synaptic sites, while ACh release simultaneously disperses clusters in non-contacted regions, thereby confining AChRs to the endplate (Trinidad et al., 2000; Xiong and Mei, 2017). During the same postnatal window in which physiological cell death occurs, each muscle fiber transitions from polyneuronal to single innervation through synapse elimination, a competitive process sensitive to the relative activity of converging terminals. Muscle fibers release trophic signals that have a permissive role in motor neuron survival, and once innervation is established, this retrograde signal is downregulated in response to evoked activity, only to be upregulated again by paralysis or partial denervation, inducing axonal sprouting (Baudet et al., 2008; Chakkalakal et al., 2010; Tovar-y-Romo et al., 2014). Among these retrograde factors, NT-4 production in skeletal muscle depends on muscle activity and is detected preferentially in slow, type I fibers; intramuscular NT-4 administration promotes sprouting of adult motor nerves, indicating that it functions as an activity-dependent trophic signal for motor neuron remodeling (Funakoshi et al., 1995). Together, these observations indicate that the NMJ is not simply the endpoint of motor axon growth, but an active retrograde signaling interface through which target-derived cues regulate motor neuron survival, synaptic refinement, and stabilization of subtype-associated functional properties.

## 6 Integrated postnatal maturation and diversification of lumbar motor neurons underlying the emergence of locomotion

Locomotor behavior emerges from a prolonged and highly coordinated maturation of lumbar spinal MNs and their associated circuits. Although the fundamental architecture of the spinal motor system is established during embryogenesis, effective locomotion depends on extensive postnatal refinement of molecular identity, intrinsic electrophysiological properties, synaptic connectivity, and non-cell-autonomous support mechanisms. Importantly, MN specification, maturation, and subtype diversification are not temporally discrete events; instead, they overlap across embryonic and postnatal development, closely paralleling the gradual acquisition of postural control and coordinated stepping.

To integrate these heterogeneous datasets across developmental stages, we summarize major milestones in lumbar MN maturation from embryogenesis through the third postnatal week (Table 1). This timeline aligns behavioral emergence (posture and stepping), molecular transitions (from specification programs toward activity- and synapse-associated modules), circuit remodeling (loss of electrical coupling, pruning of inappropriate connections, refinement of neuromuscular and premotor connectivity), and intrinsic electrophysiological maturation (declining input resistance, narrowing of action potentials, and recruitment of persistent inward currents). While the precise timing varies across species, segmental level, and experimental preparation, organizing the literature in this way highlights discrete developmental windows in which subtype identity is likely to be most plastic and molecular markers are least stable.

**Table 1:** Integrated developmental timeline of lumbar motor neuron maturation.

Functional/behavioral outcome	Molecular and genetic programs	Cellular/circuit events	Intrinsic MN properties	References
E13–E14 No organized motor output	Early MN specification TFs (ISL1/LHX3, MNX1); initiation of MN transcriptional programs	Generation of lumbar MNs from pMN domain; onset of post-mitotic migration toward columns and nascent motor pools	MNs already excitable; high input resistance, low rheobase; broad Na^+^/Ca^2+^-dependent action potentials	Altman and Bayer (1984), Chen and Chiu (1992), Jessell (2000), Lee et al. (2012), Thaler et al. (2002), Ziskind-Conhaim (1988)
E13.5–E15.5 Preparation for neuromuscular connectivity	Genes supporting axon growth and synaptogenesis	Axon growth into periphery; initial target exploration	Poor repetitive firing; immature conductance repertoire	Bravo-Ambrosio and Kaprielian (2011), Gao and Ziskind-Conhaim (1998), Suter et al. (2020), Tessier-Lavigne and Goodman (1996), Vinay et al. (2000b)
E14–E16 Activity supports circuit assembly and survival	Activity-independent developmental programs dominate	Emergence of spontaneous, bilaterally synchronized network activity	High excitability supports population bursts	Borodinsky et al. (2012), Iizuka et al. (1998), Nakayama et al. (1999), Nishimaru and Kudo (2000), Patel et al. (2022)
E15–E18 Primitive neuromuscular transmission	Synaptic organization pathways activated	Formation of early NMJs; polyneuronal innervation	Limited firing precision	Balaskas et al. (2019), Brown et al. (1976), Landmesser (1980), Patel et al. (2022), Russier et al. (2003), Sanes and Lichtman (1999), Ziskind-Conhaim (1988)
E18–E22 Transition toward functional circuits	Selection and pruning mechanisms engaged	Refinement of connectivity; onset of programmed MN cell death	Gradual stabilization of membrane properties	Hamburger (1975), Kiehn (2006), Misawa et al. (2012), Oppenheim (1991), Sendtner et al. (2000), Ziskind-Conhaim (1988)
Birth (P0) Locomotion can be evoked; posture unstable	Continued expression of early developmental genes	MNs transiently electrically coupled; inappropriate synaptic inputs persist	Diverse but immature firing patterns	Altman and Sudarshan (1975), Catela et al. (2022), Fulton and Walton (1986), Patel et al. (2022), Seebach and Mendell (1996), Walton and Navarrete (1991), Vinay et al. (2000a), Ziskind-Conhaim (1988)
P1–P7 Trunk lifting; unstable stepping; swimming possible	Regulatory shift underway: reduced embryonic TF motifs, increasing NFI family motif enrichment	Elimination of electrical coupling; early pruning of polyneuronal innervation (earlier in flexors)	Decreasing input resistance; expansion of K^+^ conductances begins	
P3–P10 Improved stepping; delayed postural control	Activity-dependent programs emerge; synaptic/receptor maturation accelerates	Faster maturation of flexor MNs vs. extensor MNs	Flexors show strong rheobase reduction; extensors lag	Gao and Ziskind-Conhaim (1998), Jablonski and Kalb (2013), Kerai et al. (1995), Manuel and Zytnicki (2019), Patel et al. (2022), Sanusi et al. (1998), Seebach and Mendell (1996), Vinay et al. (2000b)
P7–P13 Increasing coordination of limb movements	Chromatin accessibility shifts toward maturation enhancers	Growth of α and γ MNs; dendritic expansion	Improved repetitive firing; PIC recruitment	
P10–P18 Weight-bearing improves; coordinated locomotion	Upregulation of ion channel and modulatory receptor genes	Refinement of motor pool recruitment; extensor maturation progresses	Strong L-type Ca^2+^ current expression fine-tunes firing	Lee and Heckman (1998), Patel et al. (2022), Sharples and Miles (2021), Sharples et al. (2025)
P14–P21 Adult-like hindlimb movement patterns	Activity-dependent regulators dominate; muscle contraction pathways enriched	Full integration of descending control; continued synaptic refinement	Near-adult electrophysiological profile	Patel et al. (2022), Sanusi et al. (1998), Smith et al. (2017), Smith and Brownstone (2020), Westerga and Gramsbergen (1992)
P21 Mature locomotor behavior	Adult-like transcriptional profile established	Stable motor pool organization; dendritic growth continues (e.g., soleus MNs ~ 70% adult size)	Mature firing properties	
Subtype-specific features (across stages) Differential recruitment, posture, and vulnerability	Wnt7a marks γ-MNs from ~E17.5–P21; ERR2/3 detectable later; astrocytic Kir4.1 supports fast α-MNs	Delayed maturation of extensor MNs; γ-MN survival depends on muscle signals	γ-MNs smaller, highly excitable	Ashrafi et al. (1992), Burke et al. (1977), Shneider et al. (2009), Kelley et al. (2018), Khan et al. (2022), Khan and Marquardt (2024), Westerga and Gramsbergen (1992)

Summary synthesized from studies of rat and mouse lumbar MN development, including perinatal electrophysiology and circuit maturation, transcriptomic dynamics of postnatal MN maturation, and subtype-specific signaling dependencies. Timing is approximate; windows vary by species.

In rats, lumbar MNs are generated around embryonic day E13-14, after which they migrate from the pMN domain to settle into columnar and motor pool positions within the ventral horn. During late embryogenesis, axons of MNs exit the spinal cord, invade the periphery, and establish the first neuromuscular contacts. At this stage, spontaneous network activity emerges in the lumbar spinal cord and is characterized by bilaterally synchronized bursts of MN firing (Vinay et al., 2000a). MNs are already excitable by E14.5, with resting membrane potentials comparable to postnatal values, indicating that basic membrane excitability is established early. However, embryonic MNs exhibit high input resistance, low rheobase, broad Na^+^/Ca^2+^ dependent action potentials, and limited capacity for repetitive firing, reflecting an immature intrinsic conductance repertoire (Vinay et al., 2000a; Russier et al., 2003). Functionally, this early activity is thought to support circuit assembly, synaptic stabilization, and survival rather than precise motor output.

This functional immaturity is not uniform across motor pools but disproportionately affects MNs innervating postural and antigravity muscles. Extensor motor pools, which are essential for weight bearing and postural stabilization, mature later than flexor pools at both the intrinsic and circuit levels. Rodent studies demonstrate delayed refinement of extensor motor neuron intrinsic properties, slower elimination of polyneuronal innervation at extensor neuromuscular junctions, and weaker recruitment of extensor muscles during early locomotor patterns compared with flexors (Vinay et al., 2000a). Parallel observations in humans and animal models indicate that postural control emerges only after progressive maturation of descending pathways, sensory integration, and extensor-biased muscle activation, consistent with the delayed functional integration of postural motor circuits (Hadders-Algra, 2018). Together, these findings indicate that the limited postural stability observed at birth reflects a selective developmental lag of extensor motor neuron pools and their associated circuits, rather than a global immaturity of the locomotor network.

Importantly, the first postnatal week represents a critical transition away from this permissive neonatal state. Electrical coupling between MNs progressively disappears and is no longer detectable after the first postnatal week, shifting coordination toward chemical synapses and intrinsic recruitment mechanisms. Inappropriate segmental and peripheral synaptic connections are eliminated, and pruning of polyneuronal neuromuscular junction (NMJ) innervation begins, occurring earlier in flexor than extensor muscles. In parallel, descending pathways become increasingly effective, both quantitatively and qualitatively, as a result of the progressive myelination of central and peripheral axons. These changes coincide with early improvements in posture, including the ability to lift the trunk and generate unstable stepping movements.

At the level of intrinsic electrophysiological properties, lumbar MNs undergo rapid stereotyped remodeling during early postnatal life. Input resistance decreases, rheobase increases, and action potentials become narrower as potassium conductances expand, and repolarization improves (Vinay et al., 2000a; Manuel and Zytnicki, 2019). Early postnatal MNs often display delayed or transient firing patterns and poor repetitive discharge; however, by approximately P9 in rats, nearly all MNs are capable of sustained repetitive firing. This transition reflects the developmental recruitment and tuning of persistent inward currents (PICs), particularly dendritic L-type Ca^2+^ currents, which are essential for repetitive firing and gain control (Lee and Heckman, 1998; Sharples et al., 2025). Importantly, these changes are not uniform across MN populations: flexor MNs mature earlier than extensor MNs in terms of rheobase reduction, repetitive firing capability, and synaptic refinement, consistent with earlier maturation of flexor-related motor behaviors (Vinay et al., 2000a).

In parallel with electrophysiological maturation, MNs undergo extensive molecular reprogramming. Recent transcriptomic and chromatin accessibility analyses demonstrate that MNs gene expression remains highly dynamic from embryogenesis through approximately P21, after which a relatively stable adult-like profile is established (Patel et al., 2022). Early embryonic transcriptional programs that specify MN identity gradually give way to regulatory motifs associated with synaptic organization, ion channel expression, neuromodulatory signaling, and activity-dependent plasticity. By the second and third postnatal weeks, genes encoding voltage-gated ion channels, modulatory receptors, and proteins involved in synaptic transmission and muscle contraction are strongly upregulated, supporting mature firing behavior and refined motor output.

Within this global maturation trajectory, subtype diversification becomes increasingly apparent. Fast α-MN identity illustrates how molecular and electrophysiological maturation intersect. The non-canonical Notch ligand Delta-like homolog 1 (DLK1) is both necessary and sufficient to promote fast α-MN gene expression and the biophysical properties required for peak force execution, acting in part through the induction of regulatory potassium channel subunits such as Kcng4 (Müller et al., 2014). These intrinsic programs are further shaped by global shifts in chromatin accessibility as MNs transition from specification to maturation.

Taken together, the data reviewed here indicate that the emergence of mature locomotor behavior is not the consequence of a simple developmental transition. Instead, it arises from the gradual and coordinated integration of multiple processes acting across embryonic and postnatal life. Identity of MNs is progressively refined through the interplay of transcriptional and epigenetic programs, subtype-specific intrinsic electrophysiological maturation, synaptic reorganization at both central and peripheral levels, and non-cell-autonomous influences from glia, muscles, and descending circuits. Collectively, these processes converge to position each motor neuron within a multidimensional molecular-electrophysiological landscape that ultimately determines its recruitment properties, connectivity, and functional role during movement.

## 7 Transcriptomics, single-cell and spatial approaches to motor neuron subtype identity across early development

Recent transcriptomic technologies have substantially refined current views of spinal motor neuron (MN) diversity. Classical classification systems based on soma size, conduction velocity, peripheral target, anatomical position, or a limited number of markers remain useful, but they provide only partial resolution of MN heterogeneity. This limitation is particularly evident during embryonic and early postnatal life, when subtype-specific physiological and circuit properties are emerging while many adult markers are absent, broadly expressed, or developmentally unstable. Consequently, neonatal MNs remain difficult to classify using conventional approaches alone.

Single-cell and single-nucleus RNA sequencing (scRNAseq and snRNAseq) have revealed that MN identity is more complex than previously appreciated. In the adult mouse spinal cord, transcriptomic profiling identified substantial diversity among skeletal and autonomic cholinergic neurons, demonstrating that classical anatomical categories underestimate molecular heterogeneity (Blum et al., 2021). Extending this concept, targeted single-cell analysis of mammalian spinal MNs identified multiple transcriptionally distinct MN populations enriched for neuropeptides, axon-guidance molecules, adhesion proteins, and signaling regulators rather than only canonical lineage markers such as Hb9 or ChAT (Liau et al., 2023). These findings suggest that functionally relevant subtype programs may be embedded within broader MN classes and that molecular diversification may precede overt physiological specialization. More recently, integrated single-cell transcriptomic and epigenomic analysis has shown that subtype- and motor-pool-specific expression profiles can also be used to predict candidate for cis-regulatory elements. In limb motor pools, such an approach identified enhancer candidates associated with Etv4 and other column-restricted regulatory programs, indicating that recent transcriptomic datasets can inform not only MN classification but also the gene-regulatory architecture underlying pool identity (Yeo et al., 2025).

A practical methodological issue in spinal motor neuron transcriptomics is that many studies preferentially employ single-nucleus RNA sequencing instead of classical single-cell RNA sequencing. This reflects the biological properties of motor neurons: they are exceptionally large, highly polarized, and mechanically fragile cells with extensive dendritic arbors and long axons. Enzymatic tissue dissociation required for scRNA-seq can selectively damage vulnerable neurons, distort cell representation, and induce stress-response transcriptional artifacts. In contrast, snRNA-seq isolates nuclei from fresh or frozen tissue using gentler protocols, enabling improved recovery of fragile neuronal populations and compatibility with archived or postmortem human spinal cord samples (Blum et al., 2021; Cuevas-Diaz Duran et al., 2022). For these reasons, snRNA-seq has become particularly valuable in studies of adult spinal cord and motor neuron disease.

snRNA-seq, however, is not simply a superior replacement for scRNA-seq. Because nuclear RNA is sampled rather than whole-cell RNA, cytoplasmic, dendritic, and axonally localized transcripts may be underrepresented, and total gene detection per cell can be lower than in optimized scRNA-seq datasets (Bakken et al., 2018). This limitation may be especially relevant for motor neurons, whose distal compartments contain biologically important mRNAs involved in axonal maintenance and synaptic function. Accordingly, scRNA-seq can provide richer whole-cell transcriptomes when viable intact cells can be obtained, whereas snRNA-seq often provides less biased sampling of difficult tissues. These methods should therefore be viewed as complementary rather than hierarchical.

This framework is especially relevant to neonatal development. Time-series transcriptomic studies show that murine MN gene expression remains highly dynamic from embryogenesis through the first three postnatal weeks, with progressive downregulation of early specification programs and increasing expression of modules linked to synaptic transmission, ion channel composition, metabolism, and mature motor output (Patel et al., 2022). Thus, birth does not represent completion of MN differentiation; instead, neonatal MNs occupy transitional molecular states while intrinsic excitability, dendritic growth, and synaptic connectivity continue to mature. This likely explains why early postnatal MN subtypes are often easier to distinguish electrophysiologically than molecularly. A recent preprint study further supports the view that motor neuron identity remains highly dynamic beyond embryogenesis. Using transcriptomic profiling across developmental stages, Chen et al. (2025, preprint) proposed that early embryonic transcriptional programs linked to positional specification and circuit assembly progressively diminish during maturation, whereas later-emerging adult diversity is increasingly organized around genes associated with excitability, synaptic transmission, and motor output. In this framework, motor neuron identity shifts developmentally from wiring-related to firing-related molecular states, reinforcing the concept that mature subtype characteristics are assembled progressively during postnatal life rather than being fully specified during embryogenesis.

Such developmental dynamics are particularly important for α-, β-, and γ-MNs. Slow- and fast-associated α-MNs diverge postnatally in recruitment threshold, repetitive firing, and persistent inward currents, yet corresponding molecular markers often emerge gradually or incompletely. γ-MNs acquire characteristic fusimotor properties during early postnatal life, and markers such as Gfrα1, Esrrg/Err3, and reduced NeuN expression increase in usefulness as maturation proceeds (Shneider et al., 2009). In contrast, β-MNs remain poorly resolved molecularly, suggesting that neonatal single-cell datasets may provide one of the best opportunities to determine whether they represent a distinct transcriptional class or a more heterogeneous intermediate population.

An important complementary advance is spatial transcriptomics, which preserves anatomical context lost in dissociative sequencing methods. This is highly relevant for MN biology because subtype identity has historically been linked to columnar organization, motor pool clustering, and rostrocaudal position. Spatially resolved studies of the human spinal cord have shown that molecularly defined neuronal states can be mapped back onto ventral horn architecture and segmental domains, thereby linking modern molecular taxonomies with classical anatomy (Yadav et al., 2023). More recent combined snRNA-seq and spatial transcriptomic analysis of the adult human spinal cord further resolved neuronal populations into multiple transcriptional subclusters and demonstrated that these molecular classes occupy reproducible laminar and dorsoventral spatial domains, reinforcing the principle that transcriptomic identity is tightly coupled to anatomical microenvironment. The study also identified sex-associated differences in gene expression across MNs (Zhang et al., 2024). In neonatal tissue, similar approaches could help determine whether candidate molecular states correspond to flexor versus extensor pools, medial versus lateral positions, or transient maturation zones.

Human developmental datasets also provide valuable comparative context. Single-cell studies of the developing human spinal cord indicate that core patterning programs are broadly conserved across species, while developmental timing and some gene-expression features differ from rodents (Rayon et al., 2021; Andersen et al., 2023). This is particularly relevant because human postnatal maturation is prolonged relative to rodents, suggesting that subtype consolidation may follow distinct temporal trajectories.

Despite their transformative potential, transcriptomic data require cautious interpretation. Molecular clusters may reflect maturation stage, activity state, dissociation stress, or metabolic condition rather than stable subtype identity. Accordingly, transcriptomic signatures should be viewed as hypothesis-generating frameworks that require validation against electrophysiology, morphology, muscle target, and circuit embedding.

Multimodal approaches such as Patch-seq are especially promising in this regard because they combine single-cell transcriptomics with intrinsic electrophysiology and cellular morphology from the same neuron (Lipovsek et al., 2021). Although not yet widely applied to spinal MN subtype analysis, such methods could be particularly powerful in neonatal tissue, where linking candidate molecular states to recruitment properties and firing behavior is essential.

Taken together, current evidence suggests that neonatal MNs are best viewed not as miniature adult subtypes, but as populations progressing through overlapping developmental trajectories toward mature functional states. Integrating single-cell, spatial, and multimodal approaches now offers a realistic path toward resolving how stable MN subtype identity emerges during early postnatal life.

## 8 Summary and perspectives

Early postnatal life is a critical period during which spinal motor neurons (MNs) undergo rapid refinement of intrinsic excitability, synaptic connectivity, and recruitment properties. Although functional heterogeneity is already evident, stable molecular identification of emerging α-, β-, and γ-subtypes remains incomplete, particularly during the neonatal period when many classical markers are developmentally unstable or only partially informative.

The evidence reviewed here supports a model in which MN subtype identity is progressively consolidated after birth through interactions between embryonic specification programs, activity-dependent maturation, target-derived signals, and evolving transcriptional states. In this view, neonatal MNs occupy dynamic developmental states that gradually converge onto mature functional phenotypes rather than representing fully specified adult classes present at birth.

This framework also has important translational implications. Selective vulnerability is a defining feature of motor neuron disorders such as amyotrophic lateral sclerosis (ALS) and spinal muscular atrophy (SMA), in which some MN populations degenerate early whereas others remain comparatively resistant. Historically, these differences have been described mainly at the level of large motor pools or muscle groups, because molecular tools lacked the resolution to distinguish finer neuronal subtypes. Recent single-cell studies suggest that previously unrecognized MN diversity may underlie these susceptibility patterns. For example, transcriptionally defined subpopulations enriched for Calb1 or mGluR5 have been associated with relative resistance to degeneration, raising the possibility that intrinsic calcium buffering, metabolic programs, or pro-survival signaling pathways contribute to subtype resilience (Liau et al., 2023).

At the same time, disease-associated transcriptional “MN states” identified by single-cell profiling should be interpreted cautiously. Stress-response, mitochondrial, inflammatory, or regenerative signatures may reflect convergent injury responses rather than pre-existing vulnerable subtypes (Schweingruber et al., 2025). Accordingly, the most informative path forward will be to integrate transcriptomic states with electrophysiology, morphology, spatial localization, and developmental lineage in order to distinguish true subtype-selective degeneration from secondary pathological remodeling.

Recent single-cell, spatial, and multimodal approaches now provide the tools to resolve these trajectories with unprecedented precision. Future work should prioritize identifying temporally robust subtype markers during early postnatal life, defining how developmental state influences later disease risk, and determining whether protective molecular programs can be therapeutically reactivated. Understanding how MN identity is assembled during neonatal maturation may therefore prove essential not only for basic motor system biology, but also for the development of subtype-informed therapies for ALS, SMA, and related neuromuscular disorders.
